# Electrospun Membranes Designed for Burst Release of New Gold-Complexes Inducing Apoptosis of Melanoma Cells

**DOI:** 10.3390/ijms23137147

**Published:** 2022-06-27

**Authors:** Liberata Guadagno, Marialuigia Raimondo, Luigi Vertuccio, Erwin Pavel Lamparelli, Maria Camilla Ciardulli, Pasquale Longo, Annaluisa Mariconda, Giovanna Della Porta, Raffaele Longo

**Affiliations:** 1 Department of Industrial Engineering, University of Salerno, 84084 Fisciano, Italy; mraimondo@unisa.it; 2 Department of Engineering, University of Campania “Luigi Vanvitelli”, 813031 Aversa, Italy; luigi.vertuccio@unicampania.it; 3 Department of Medicine, Surgery and Dentistry, University of Salerno, 84081 Baronissi, Italy; elamparelli@unisa.it (E.P.L.); mciardulli@unisa.it (M.C.C.); gdellaporta@unisa.it (G.D.P.); 4 Department of Chemistry and Biology, University of Salerno, 84084 Fisciano, Italy; plongo@unisa.it; 5 Department of Science, University of Basilicata, 85100 Potenza, Italy; annaluisa.mariconda@unibas.it; 6 Interdepartment Centre BIONAM, Università di Salerno, 84084 Fisciano, Italy

**Keywords:** electrospinning, melanoma topical treatment, drug release

## Abstract

Two non-commercial metallic Au-based complexes were tested against one of the most aggressive malignant melanomas of the skin (MeWo cells), through cell viability and time-lapse live-cell imaging system assays. The tests with the complexes were carried out both in the form of free metallic complexes, directly in contact with the MeWo cell line culture, and embedded in fibers of Polycaprolactone (PCL) membranes produced by the electrospinning technique. Membranes functionalized with complexes were prepared to evaluate the efficiency of the membranes against the melanoma cells and therefore their feasibility in the application as an antitumoral patch for topical use. Both series of tests highlighted a very effective antitumoral activity, manifesting a very relevant cell viability inhibition after both 24 h and 48 h. In the case of the AuM1 complex at the concentration of 20 mM, melanoma cells completely died in this short period of time. A mortality of around 70% was detected from the tests performed using the membranes functionalized with AuM1 complex at a very low concentration (3 wt.%), even after 24 h of the contact period. The synthesized complexes also manifest high selectivity with respect to the MeWo cells. The peculiar structural and morphological organization of the nanofibers constituting the membranes allows for a very effective antitumoral activity in the first 3 h of treatment. Experimental points of the release profiles were perfectly fitted with theoretical curves, which easily allow interpretation of the kinetic phenomena occurring in the release of the synthesized complexes in the chosen medium.

## 1. Introduction

Cancer is one of the main diseases of this century [1]. Given the existence of numerous types of tumors, different possible therapies are envisaged, depending on the stage of progress of the pathology [2,3,4].

In the drug delivery field, the need for new more effective therapies has led to scientific progress mainly in two directions: the development of new active molecules and the development of innovative systems for drug administration [5,6,7,8,9,10,11].

Combining these two approaches, the present study proposes a highly innovative material with synthetic antitumoral gold-based complexes, developing a cutting-edge wearable patch-like device that demonstrates excellent performance against melanoma.

**Figure 1:**
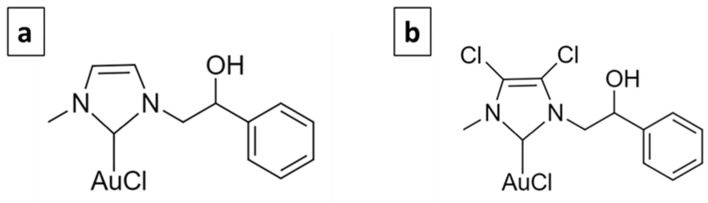
AuL20 (**a**) and AuM1 (**b**).

The use of metal-based molecules for cancer treatment is widely accepted, from cisplatin to titanocene dichloride, and they are generally based on Pt, Au, Ag, Rh, etc. [12]. The complexes selected for this study are based on gold. Gold has been used since ancient times to treat various diseases. The first scientific evidence on the therapeutic efficacy of gold dates back to the 1920s and relates to the compound K[Au(CN)_2_], whose antituberculous activity has been clinically tested [13]. Currently, Auranofin (1-thio-β-D-glucopyranosatotriethylphosphine gold-2,3,4,6-tetraacetate) is used in the treatment of rheumatoid arthritis and has also been tested in experiments carried out to evaluate its antitumor activity, giving important results [14]. Its target is mainly represented by the mitochondrial enzyme thioredoxin reductase (TrxR) [15]. Gold complexes with N-heterocyclic carbene (NHC) ligands have the ability to potently inhibit TrxR and decrease tumor cell proliferation [16,17]. Furthermore, they interfere with the metabolism of tubulin and/or actin and therefore play an important role in regulating the dynamics of the cytoskeleton [18,19,20,21,22]. Previously, two NHC-ligated gold-based molecules, namely AuL20 and AuM1 (Figure 1) [19,22], were identified, which showed remarkable antitumor activity.

In the present study, the applicability of Au-complexes to promote topical drug delivery is investigated. Different percentages of the two complexes were loaded in electrospun polymeric matrices. Membrane’s design and production are tailored to make them easily applied for topical treatments, providing an efficient release directly in the zone needing for the treatment.

In particular, electrospun membranes produced were tested against human malignant melanoma, the MeWo cell line, evaluating their cytotoxicity. MeWo represents one of the most aggressive forms of melanoma tumor; these cells are harvested from a metastatic site in the lymph node tissue. No effective treatment for metastatic melanoma exists, hence currently, an intense effort for new drug evaluation is strongly desirable [23].

Among the numerous techniques able to produce transdermal drug delivery systems, electrospinning was chosen, mainly for two reasons. On the one hand, for the possibility of producing films composed of small-sized polymer fibers (50–5000 nm), that can well mimic the human tissue and, hence, is also particularly suitable for wound dressing. In this perspective, these patches could be easily applied, for example, before or after surgery, using them for topic chemotherapy and protection of the treated areas [24]. On the other hand, the electrospinning process is suitable for processing biodegradable and biocompatible materials in several configurations [25], and is ideal for materials applicable in the biomedical field [26]. In this study, polycaprolactone (PCL) was chosen, since it allows for obtaining a mat composed of fibers with reduced average dimensions, biocompatibility, biodegradability and good mechanical performance [27,28]. PCL-based electrospun membranes were successfully functionalized with different types of filler, from active molecules to nanoparticles [29,30,31,32,33], and tested against other aggressive skin cancer [34,35].

In the current paper, PCL electrospun membranes, loaded with different percentages of AuM1 and AuL20, are produced, and the process conditions are described. The cytotoxicity of the complexes alone (free) and the functionalized membranes are tested against MeWo cells. By Field Emission Scanning Electron Microscopy (FESEM) and Atomic Force Microscopy (AFM) analyses, the net morphology, the fiber dimensions, and the distribution of the complexes in the nanofibers are studied. Eventually, the obtained release profiles are modeled.

## 2. Experimental Section

### 2.1. Materials

Poly(ε-caprolactone) (PCL-CAS N° 24980-41-4; Molecular weight 80,000 Da) was purchased by Perstorp (Warrington, UK). Dimethylformamide (DMF-CAS 68-12-2) was purchased from Sigma Aldrich (Burlington, VT, USA). Acetone was purchased from Aldrich Chemical Corporation (St. Louis, MO, USA). Chloroform was purchased from Carlo Erba (Cornaredo, Italy). Phosphate buffered saline (PBS) (pH 7.3) was purchased by Oxoid (Basingstoke, UK).

Au-complexes were prepared according to the procedure reported by Mariconda et al. [36]. AuL20 was obtained using styrene-oxide, which by the opening of the epoxy-ring, reacts with imidazole giving the monoalkylated product N-methyl, N-[(2-hydoxy-2-phenyl) ethyl]-imidazolium iodide. The second nitrogen atom is methylated using CH_3_I, producing the racemic mixture of the salt. It was reacted with silver oxide (Ag_2_O) in an inert nitrogen atmosphere. In these conditions, the silver oxide deprotonates the cationic carbon, giving the corresponding Ag–NHC complex.

The silver complex is reacted with chloro-(dimethylsulfide)-gold(I) [(CH_3_)_2_SAuCl] in dichloromethane. The reaction was left for 1 h at room temperature, then the mixture was filtered, and the solvent removed in vacuo. The obtained solid AuL20 was characterized by ^1^H NMR and ^13^C NMR, mass spectrometry, and elemental analysis. The same procedure was applied in the synthesis of AuM1 using 4,5-dichloroimidazole instead of imidazole. Both complexes were obtained in the form of solid powders. The complete analysis (^1^H NMR, ^13^C NMR, ESI-MS, CHN) of the synthesized complexes is provided in the Supplementary Materials (Figures S1–S6).

The analysis of the hydrolytic stability of the complexes, carried out at 40 °C in an aqueous solution of DMSO-d6 at 10% on these complexes, by means of ^1^H NMR spectroscopy, demonstrated that these complexes are particularly stable. In fact, their spectra recorded after 24 h are practically identical to those recorded at zero time (an amount of more than 97% of complexes’ results not hydrolyzed). This ensures that the NHC ligands are ancillary ligands and therefore stably bound to the metal centers [19].

### 2.2. Preparation of Solutions for Electrospinning Membrane

PCL pellets were added to Acetone/DMF mixture (3:1 in volume) at 11 wt.%. Different amounts of complexes AuL20 and AuM1 were added in the PCL solution: 0%, 1%, and 3% by weight of active complex obtaining the samples named with the acronyms PCL, 1%AuL20, 3%AuL20, 1%AuM1 and 3%AuM1. The solutions were kept under magnetic stirring at 40 °C for 24 h to obtain homogeneous solutions.

### 2.3. Electrospinning Procedure

The polymeric solutions were electrospun by the climate-controlled electrospinning equipment (EC-CLI by IME Technologies, Spaarpot 147, 5667 KV, Geldrop, The Netherlands). Each solution was loaded in a syringe of 5 mL and fed to a 0.8 mm diameter needle connected to a power supply. The flow was ejected by the needle in the climate room in the presence of a strong electric field that allows the spinning of the polymer. It was necessary to vary the process conditions among the various membranes depending on the spinnability of the solution; however, the room conditions were set at a temperature of 25 °C and the relative humidity at 35%. The other process parameters are reported in Table 1 for all the prepared and tested membranes. It is worth noting that samples loaded with a higher percentage of complexes required higher values of the Electrical Potential difference to obtain membranes with a good distribution of the morphological parameters, as discussed in the section “Morphological and structural characterization”. Table 1 shows only the electrospinning parameters employed for the membranes optimized from the point of view of the electrospinning process and subsequently tested for evaluating the anticancer effectiveness.

### 2.4. Sample Preparation and Sterilization Protocol

Free metallic complexes were solubilized in dimethyl sulfoxide (DMSO) and diluted in Dulbecco’s Modified Eagle’s Medium (DMEM) high glucose (GIBCO, Invitrogen, Walthan, MA, USA) at a final concentration of 1, 5, 10 and 20 μM, for cell treatments.

For adhesion culture, not functionalized and functionalized PCL membranes were cut to obtain a cycle shape of 15 mm of diameter and then they were dipped in 70% ethanol and washed twice in sterile PBS 1X. Samples were dried for 24 h under a laminar flow cabinet.

Silicon rings were cut using a hollow cutter (outer diameter: 14 mm; inner diameter: 11 mm) and sterilized in 70% ethanol. After ethanol evaporation, silicon rings were stuck on PCL membranes using non-corrosive silicon rubber and left to dry overnight. Samples were dipped in 70% ethanol, exposed to UV rays for 5 min on both sides and then used for cell seeding.

### 2.5. Cell Culture in Adhesion

To test the cytotoxicity of free metallic complexes, MeWo (ATCC^®^, HTB-65TM; P22) were seeded in 96-well plates at a density of 100.000 cells/mL. Cells were cultured in DMEM high glucose (GIBCO, Invitrogen, Walthan, MA, USA) containing 10% Fetal Bovine Serum (Gibco^TM^, Walthan, MA, USA), 1% Penicillin/Streptomycin (Corning, Manassas, VA, USA), and 1% Glutagro^TM^ (Corning, Manassas, VA, USA) at 37 °C in a 5% CO_2_ atmosphere. After 24 h, different concentrations of each complex (1, 5, 10 and 20 µM) were added to their respective wells and incubated for 24 h and 48 h.

To test the cytotoxicity of functionalized PCL membranes, MeWo (ATCC^®^, HTB-65^TM^; P27) were seeded within silicon rings on PCL membranes, to prevent cells flushing from the membrane area, at a density of 30.000 cells/cm^2^. Samples were cultured in DMEM high glucose (GIBCO, Invitrogen, Walthan, MA, USA) supplemented with 10% Fetal Bovine Serum (Gibco^TM^, Walthan, MA, USA), 1% Penicillin/Streptomycin (Corning, Manassas, VA, USA) and 1% Glutagro^TM^ (Corning, Manassas, VA, USA) at 37 °C in a 5% CO_2_ atmosphere [37,38,39].

### 2.6. Cell Viability Assay

For suspension culture, after 24 h and 48 h, 0.5 mg/mL of 3-(4,5-Dimethylthiazol-2-yl)-2,5-diphenyl-tetrazolium bromide (MTT) was directly added in the culture medium and incubated for 4 h, protecting the plate from the light. Then, the supernatant was removed and 100 µL of 100% DMSO was added to solubilize formazan crystals.

The absorbance was measured at 490 nm using a microplate reader (Infinite F200 PRO, Tecan Group Ltd., Männedorf, Switzerland). Cell viability was calculated as the percentage of the control group, considered as 100%. The percentage viability of cells was calculated according to Equation (1):

(1) $$\%Cell\;viability=\frac{Abs\;of\;sample-Abs\;blank}{Abs\;of\;control-Abs\;blank}\times 100$$

### 2.7. Time-Lapse Live-Cell Imaging System Assay

Specific MeWo culture was performed using a Time-lapse Live-Cell Imaging System formed by a Bold Line Top Stage Incubator for 35 mm Petri dishes (H301-T UNIT BL; Okolab S.r.l., Pozzuoli, Italy), which allows the acquisition of the same images along the culture time in a fixed culture point mapped by the fully-automated stage. The incubator has independent control of gas (CO_2_/O_2_), humidity and temperature and assures an environment with 37 °C of temperature and 5% of CO_2_ atmosphere. The system allows acquisition in brightfield and fluorescence. All images of different points within the culture chamber were reached automatically, using Olympus IX83 time-lapse microscope by motorized stage and CCD monochrome camera (mod. XM10, Olympus, Tokyo, Japan), and with all operations under the control of the X-Excellence advanced live-cell imaging software (rel. 2.0, Olympus Inc., Hamburg, Germany). Cells’ images were captured in brightfield using a 10× objective every 4-h intervals along the 48 h of culture, and further details on cells’ characterization were reported elsewhere [40]. The related videos reported in additional data were generated with windows movie maker software (Version 2.0, Microsoft) starting from the acquired frames.

### 2.8. Statistical Analysis

Results from multiple experiments are presented as mean ± standard deviation (SD). Statistical analysis was performed using the two-tailed independent Student’s *t*-test for comparisons of two independent groups. *p* values less than 0.05 were accepted as significant [41]. All statistical analysis was conducted using GraphPad Prism software (6.0 for Windows).

### 2.9. Morphology Analysis

Morphological analysis was performed by using Field emission scanning electron microscopy (FESEM). The samples were coated with a thin gold layer before the FESEM analysis. By the acquired images, geometrical information and the size distribution of the nanofibers were obtained. The analysis of the fiber distribution was performed by using ImageJ, considering at least 150 fibers. The analysis of the pore size was performed by using the software MATLAB (Natick, MA, USA) following the procedure reported in the literature [31,42]. The images were collected with a FESEMLEO1550VP microscope (Carl Zeiss SMT AG, Oberkochen, Germany).

For Energy Dispersive X-ray Analysis (EDX) investigation, a FESEM LEO1525 microscope (Oberkochen, Germany) equipped with an EDX detector was used. EDX maps were obtained after sputtering the samples with a thin coating of chromium. This investigation was performed for AuM1-loaded membranes.

**Figure 2:**
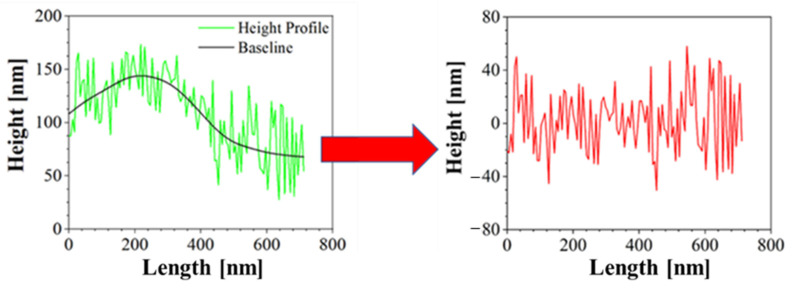
Correction of Height profile for roughness evaluation.

Moreover, Atomic Force Microscopy (AFM) was performed to better characterize the morphology of the samples. AFM is a technique able to recreate a topographic map of the sample surface by exploiting the interactions between the tip and the sample surface. By acquiring information on the deflection of the cantilever through a laser, it is possible to obtain morphology accurate data on the surface of the sample. In this case, Bruker NanoScope V multimode AFM (Digital Instruments, Santa Barbara, CA, USA) was used, in tapping mode and ambient atmosphere. The tip used has a nominal spring constant of 20–100 N/m, resonance frequencies of 200–400 kHz and a tip radius of 5–10 nm. This investigation was performed both for blank PCL and AuM1-loaded membranes. The height profiles along the fibers were acquired using NanoScope Analysis 1.40 Software (Bruker Corporation, Billerica, MA, USA), and were elaborated via OriginPro software (OriginLab Corporation, Northampton, MA, USA) to consider the height profile without being affected by the fiber slope in the chosen region. The data elaboration is displayed in Figure 2.

In this way, the average surface roughness (*R_a_*) and the root mean square roughness parameter (*R_q_*) were evaluated for the various membranes, according to (2) and (3):

(2) $$R_{a}=\frac{1}{l_{r}}\int_{0}^{l_{r}}\left|z\left(x\right)\right|\times dx$$

(3) $$R_{q}=\sqrt{\frac{1}{l_{r}}\;\int_{0}^{l_{r}}z{\left(x\right)}^{2}dx}$$

where *l_r_* is the length of the line, *z* is the height and *x* is the position.

### 2.10. Structural Characterization

Structural characterization of electrospun membranes was performed by X-ray Diffraction (XRD) measurements using the diffractometer Bruker D8 Advance diffractometer (Bruker Corporation, Billerica, MA, USA) operating at 35 kV and 40 mA. The analysis was performed in an angle range (2θ) of 10–80°. The spectra presented in the section of the “Results” are shown between 15–35° because the curves are flat out of this range. This investigation was performed both for blank PCL and AuM1-loaded membranes.

Crystallite sizes were determined using Scherrer Equation (4):

(4) $$\tau =K\times \frac{\lambda }{\beta \times \cos\left(\theta \right)}$$

where *τ* is the mean size of the crystallite, *λ* is the wavelength of the X-ray source (0.1542 nm), and *β* is the width of the peak at half maximum intensity, whereas θ is the diffraction angle. The data were analyzed with the same methodology reported by Naddeo et al. [43].

The crystallinity of the sample was obtained according to Sownthari et al. [44] by deconvoluting the spectra and considering the crystalline and the amorphous areas under the diffractometric curve profile, according to Equation (5):

(5) $$X_{c}=\frac{A_{crystalline}}{A_{crystalline}+A_{amorphous}}$$

### 2.11. Release Profiles

Samples of membranes of 1 cm diameter were placed in PBS and stirred at 300 rpm in an orbital shaker. The release medium was taken at a fixed time and then replaced with fresh PBS. Drug release kinetics were monitored by using a Spectrometer UV-2401 PC (Shimadzu, Kyoto, Japan). The tests were performed using rectangular plates with an exposed area of about 3 cm^2^ and 1 cm of the light path.

Considering the chemical structure of AuM1 and AuL20, the phenyl group was tracked in the release medium to monitor drug release. However, it is known that benzene absorbs UV radiation causing a π→π* at 180 nm, 203.5 nm and 254 nm [45,46]. In this case, the 254 nm peak was monitored for 5 days. By tracking the absorbance of phenyl group of known quantities of AuM1 and AuL20, the calibration curves are well described by the Lambert–Beer Equation (6).

(6) A=ε×c×l

where *A* is the absorbance, *ε* is the absorptivity of the complex, *c* is the concentration of the solution and *l* is the light path length.

Table 2 reports the *ε* values for the complexes.

**Figure 3:**
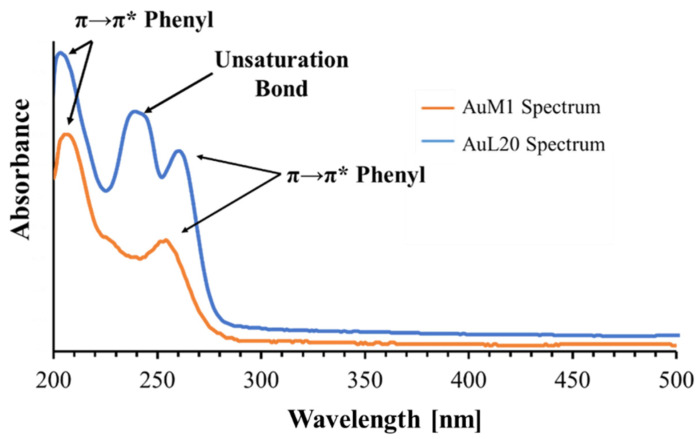
Spectra of AuM1 and AuL20.

The peak monitored for AuL20 is slightly shifted from 254 nm (typical peak of the phenyl group) to 260 nm; whereas the peak value of AuM1 complex is detected around the expected value (252 nm). The spectra are reported in Figure 3.

Observing the spectra, π→π* transitions around 254 nm and around 200 nm caused by phenyl are evident for both complexes dissolved in the solution. However, for AuL20, a third peak is registered around 240 nm. Since AuM1 shows the presence of two chlorines on the backbone compared to the AuL20 chemical structure, it is reasonable that Cl atoms attract electronic density given its high electronegativity, decreasing and shifting the intensity of the π→π* transition of the electrons involved in the double bond of the two carbonium (see the shoulder at 226 nm visible in AuM1 spectrum).

In AuL20, where there are two hydrogens instead of Cl, the transition is allowed and it is very evident in the spectrum. π→π* double bond transition in literature can be found in very different ranges of the UV-vis spectrum, from 184 nm for ethylene to over 400 nm for molecules such as *β*-carotene [45].

For release profiles fitting, a statistical approach was followed by using a modified Weibull model (7) recently proposed in literature [31]:

(7) $$\frac{m}{m_{0}}=\theta \times [1-\exp\left(-\frac{1}{A_{1}}\times t^{b_{1}}\right)]+\left(1-\theta \right)\times [1-\exp\left(-\frac{1}{A_{2}}\times t^{b_{2}}\right)]$$

where *m* is the substance amount released at a certain time, *m*_0_ is the total substance amount in the sample (evaluated by weighting the sample and knowing the complex percentage in the membrane), *A*_1_, *A*_2_, *b*_1_, *b*_2_ and θ are kinetic constant parameters, *t* is the time.

In general, the two parts of the model can describe two contributes, that are usually considered the Case II transport and the Fickian Diffusion, in agreement with other variations of the Weibull Model [47]. In particular, θ defines which mechanism is more relevant, whereas *A*_1_, *A*_2_, *b*_1_ and *b*_2_ define the kinetic of each mechanism.

Moreover, the first-order kinetic and Ritger–Peppas models were used together to explain the mechanisms that control the release behavior following the approach of Wu et al. [48]. The models used are reported below in Equations (8) and (9):

(8) $$\frac{m}{m_{0}}=1-\exp\left(-a\times t\right)$$

(9) $$\frac{m}{m_{0}}=k\times t^{n}$$

where *a* and *k* are kinetic constants and *n* is a descriptive parameter of the diffusive release [49].

## 3. Results and Discussion

### 3.1. Anticancer Activity of Complexes and Functionalized Membranes

#### 3.1.1. Viability Data on Free Metallic Complexes

**Figure 4:**
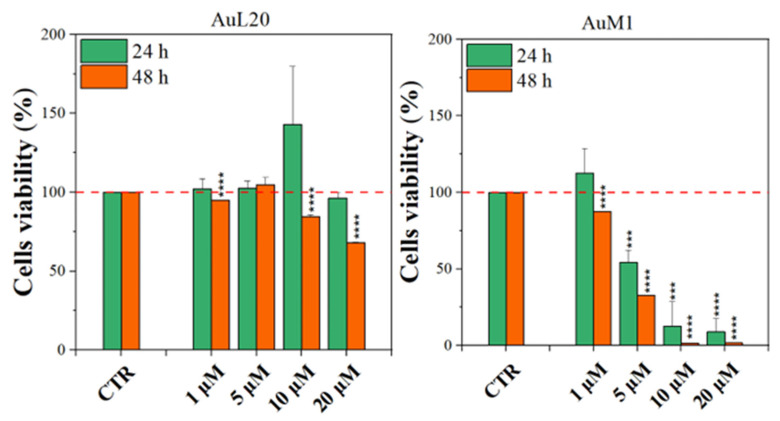
Cell viability of MeWo cells treated with different concentrations of each metallic complex for 24 and 48 h by the MTT assay. The experiments were analyzed by two-tailed Student’s *t*-test, *** *p* < 0.001 and **** *p* ≤ 0.0001.

Before testing the PCL membranes, the cell viability was assessed using the MeWo cell line cultured with different concentrations of the free metallic complexes. From the reported histograms (see Figure 4), it emerged that the two complexes are able to significantly reduce cell viability. Of note, the highest tested concentration of complexes (20 μM) was the most effective in inducing cell mortality. Moreover, the AuM1 complex demonstrated the best dose and time-dependent effect, suggesting that it could be considered the most promising antiproliferative compound for these cells. Concerning the 10 μM concentration of AuL20, we found an apparent increase in cell viability at 24 h that could be due to a possible interaction with some receptors involved in cell proliferation, even if the data demonstrated an extremely large standard deviation and were not statistically significative. Furthermore, these results were not corroborated by a similar trend at 48 h of the 10 μM treatment, suggesting that it may be only a transient and not significant effect. On the other hand, the cell viability inhibition is particularly evident after 48 h for the concentration of 20 μM for both complexes that significantly reduced cell viability. Particularly, the complex AuM1 resulted in the most effective with a specific dose-response trend and also seemed to be characterized by an excellent selectivity, with respect to non tumoral cell lines. Detailed data on this aspect will be published in a forthcoming paper currently in preparation.

These results suggest that metallic complexes, such as AuM1, could be particularly interesting for further investigation in the fight against these aggressive melanomas, especially considering the fact that they have displayed a significantly higher cytotoxic activity than chemotherapic commonly used for melanoma treatments, such as Dacarbazine (not reported here).

**Figure 5:**
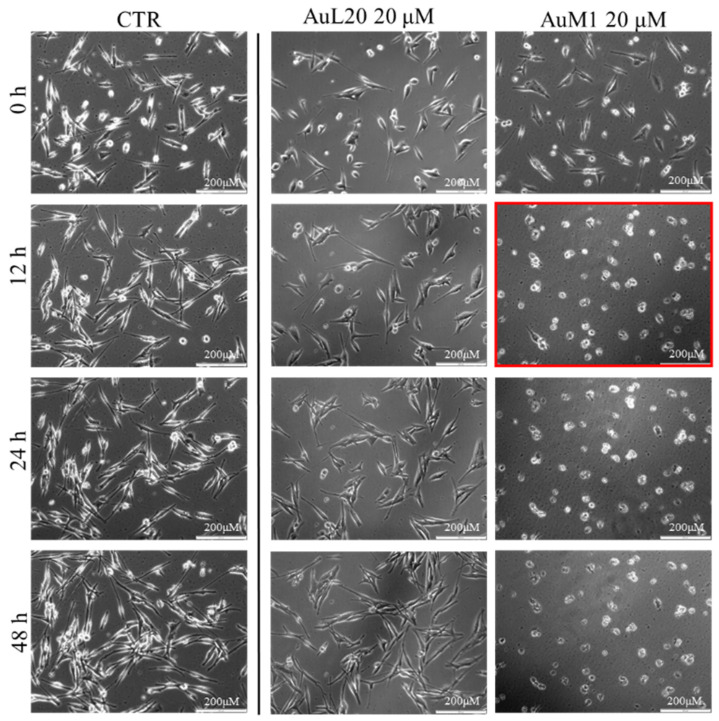
Cell viability of MeWo cells treated with 20 µM of each metallic complex for 48 h of cells monitored by Time-lapse Live-Cell Imaging System assay.

To further investigate the effective activity of both complexes and to understand whether there was a time-dependence on their activity, a culture was performed on an incubator under a camera acquiring time-lapse frames of the specific and previously fixed areas of culture, and the related videos were collected. The time-lapse cell imaging protocol was adopted to monitor the time necessary for cells to die when treated with 20 μM of each metallic complex. The time-lapse images acquired are reported in Figure 5. The time-lapse videos are reported in the additional data section on the paper site.

The time-lapse acquisitions confirmed that the AuM1 complex was the fastest and the most active complex in inhibiting cells’ survival. Indeed, just after 12 h, apoptosis occurred, as evidenced by the shape of the cells that suddenly changed into a spherical structure (see panel highlighted in red in Figure 5).

#### 3.1.2. Viability Data of Functionalized Membranes

**Figure 6:**
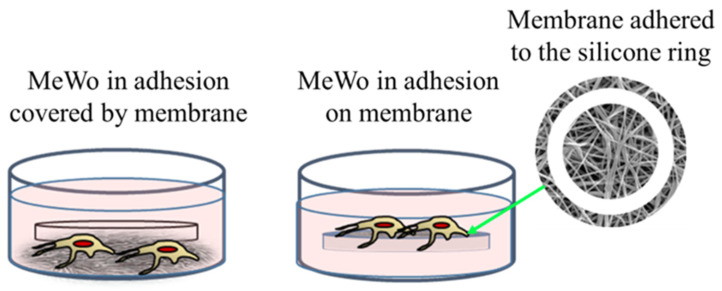
Cells’ culture and membrane: options tested.

The membrane experiments turned out to be more complicated than expected. Indeed, preliminary cultures were performed with membranes of 200 μm of thickness and loaded with 1% of AuM1 complexes using the selected MeWo cells adhered to the conventional flask. However, simply inserting the membrane samples within the culture with adherent cells was not successful. Indeed, several membrane samples precipitated on the bottom of each well (see Figure 6) preventing almost all cells survival; however, that was found mainly attributable to the limited gases exchanges (mainly oxygen) and not properly to the presence of complexes incorporated in the membrane fibers. Indeed, PCL membranes, without complexes, also demonstrated high toxicity (data not shown here). Therefore, the results of this first adopted methodology were considered unreliable data. However, because the functionalized membranes were designed to be used as patches for topical application on solid tumors, the MeWo cell line was still considered the best option to be tested. Therefore, a new methodology was adopted by seeding the cells directly on the membrane surface, thanks to a silicone ring, in order to avoid cells flushing out from the membrane to the bottom of the well plate, as illustrated in Figure 6. After adopting this methodology, statistically significant data were collected due to proper metabolites and oxygen exchanges as well as the prevention of cells’ losses; therefore, the reduced vitality was considered only due to the membrane toxicity.

**Figure 7:**
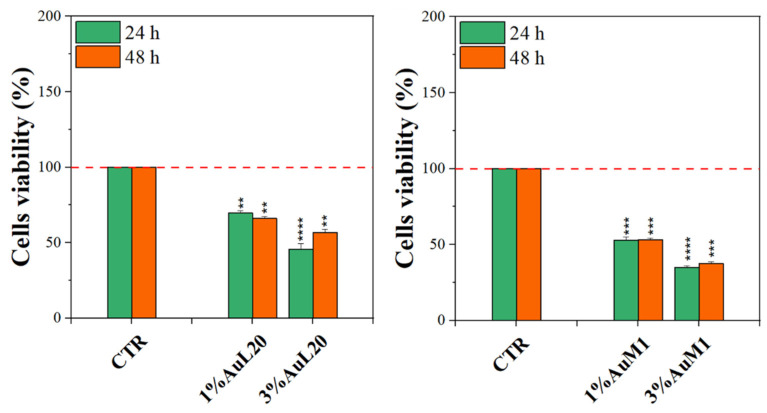
MTT assay on MeWo cells when cultured on 1% and 3% functionalized PCL membrane surface for 24 h and 48 h. The experiments were analyzed by two-tailed Student’s *t*-test, ** *p* < 0.01, *** *p* < 0.001 and **** *p* ≤ 0.0001.

Complexes-filled PCL membranes at concentrations of 1% and 3% were tested, and the resulting viability data are shown in Figure 7. All membranes functionalized with the complexes were able to decrease cell viability, both at 24 and 48 h of culture, in comparison to not functionalized membranes (CTR sample). Concerning 1% of functionalized PCL membranes, cell viability exhibited a reduction of about 50%; whereas the cytotoxic effect was found stronger when PCL membranes were functionalized with 3% of metallic complexes, where the percentage is mainly lower than 50%. Among the two tested complexes, AuM1 was found to be the most promising and statistically significant compound in reducing cell viability up to 40% when loaded at 3% within PCL membranes, in agreement with the results of the tests performed using the free complexes, which also manifest a dose-dependent cytotoxic behavior against MeWo cells. All data also suggested that the complexes loaded in the membrane were suddenly released in order to reach the proper cytotoxic concentrations required to promote cells’ apoptosis. However, further indications on complex release profiles were demonstrated and discussed in the following.

The same tests performed with the membranes loaded with the complexes were carried out using unloaded membranes to test the effect of the PCL alone on the viability of the cells. The results are exhibited in Figure S7 of Supplementary Materials. It is well evident that no cytotoxic effect is observed. Actually, after 48 h, the peculiar morphology of the membrane seems even to favor the growth of the tumoral cells, as expected.

### 3.2. Morphological and Structural Characterization of Membranes

#### 3.2.1. Morphological Characterization

**Figure 8:**
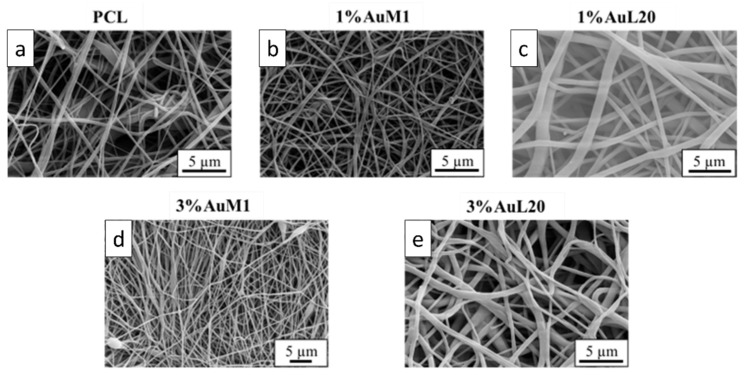
FESEM of the membranes: (**a**) PCL; (**b**) 1%AuM1; (**c**) 1%AuL20; (**d**) 3% AuM1; (**e**) 3%AuL20.

The membranes tested to evaluate the action on the proliferation of MeWo cells were deeply analyzed from the morphological and structural points of view. FESEM images of all tested membranes are shown in Figure 8, where continuous fibers without visible defects (broken fibers, debris, etc.) are well evident.

**Figure 9:**
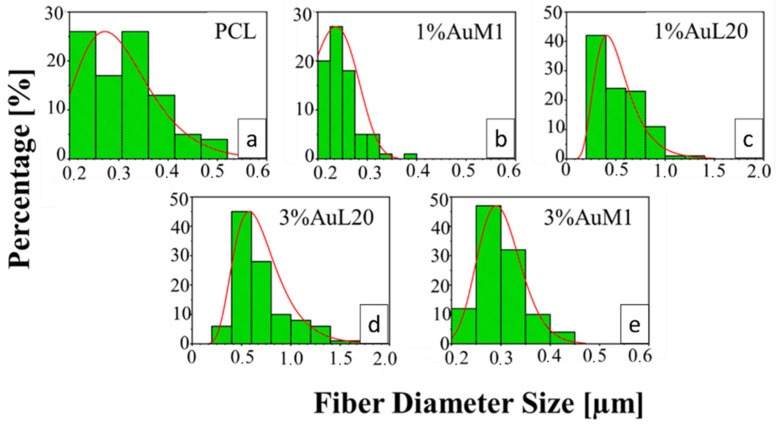
Fiber diameter distribution of: (**a**) PCL; (**b**) 1%AuM1; (**c**) 1%AuL20; (**d**) 3%AuL20; (**e**) 3%AuM1.

All the membranes show an average fiber diameter below 1 μm, as confirmed by the fiber diameter distribution in Figure 9. Although quite homogeneous membranes were obtained, Figure 9 evidences a slight dependence of the dimensional distribution of the fibers on the load and nature of the complex, most likely mainly due to the different parameters set for the electrospinning process.

The average fibers’ diameter falls between 200 and 700 nm, demonstrating monomodal distribution. Moreover, for PCL, 1%AuM1, 3%AuM1 a narrow fiber distribution is evident, with an average fiber diameter below 300 nm. By MATLAB processing of the images, the pore size distribution was acquired. The results are briefly presented in Table 3. 

**Figure 10:**
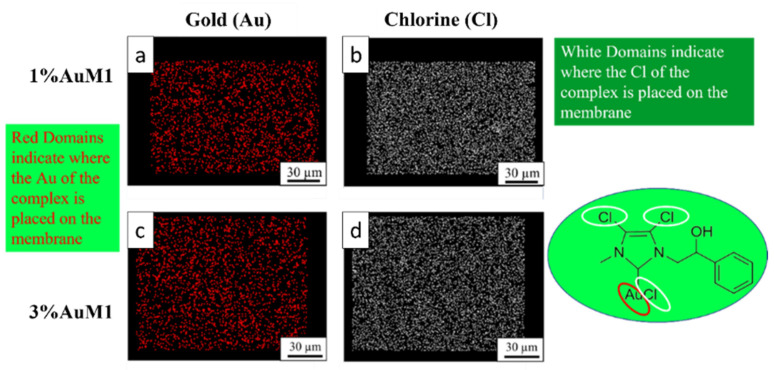
EDX images of 1%AuM1 and 3%AuM1 samples of gold (**a**,**c**) and chlorine (**b**,**c**), respectively. Images (**a**,**b**) refer to samples with 1% of the complex, whereas (**c**,**d**) to samples with 3% of complex.

Given the higher antitumoral efficiency demonstrated by AuM1-membranes, a further morphological analysis was performed to investigate the distribution of the AuM1 complex and its location in the fiber membranes. By EDX analysis, the distribution of Au-complexes was investigated, tracking Au and Cl on the membrane surface. The images of EDX analysis with Au and Cl tracked are shown in Figure 10.

Small molecular aggregates of Au-complexes (red zones) are well distributed on the entire surface of the membranes, proving that the electrospinning process is a valid choice to integrate synthesized complexes in patch-like structures ensuring a good distribution along the nanofibers of the mat membrane. Chlorine response is denser than Au since AuM1 structure is based on three atoms of Cl and one of Au. As expected, the tracing of the chlorine leads to the same conclusions about the distribution of the complex in the fibers.

**Figure 11:**
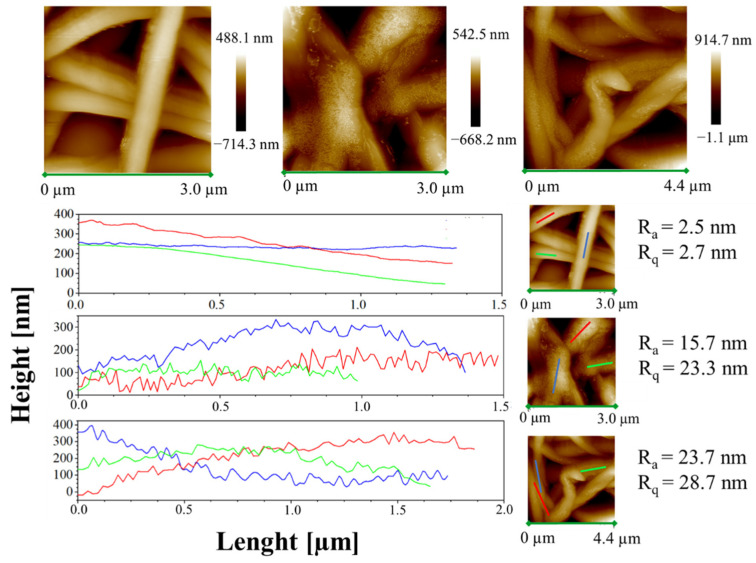
Height AFM images and profiles of the height variations along the lines parallel to the axis fibers for the samples PCL, 1%AuM1 and 3%AuM1.

Moreover, height AFM images, shown in Figure 11, evidence that a large amount of the complex aggregates is segregated on the nanofiber surface along the fibers, forming very small aggregates externally attached to the PCL nanofibers.

This phenomenon likely shows that the complexes are loaded above the solubility limit in the polymer. As reported by Natu et al. [52], in these cases the drugs crystallize on the surface of the nanofibers, causing a relevant burst effect. The low solubility of AuM1 in PCL is probably due to the hydrophilicity of the complexes (-OH group in the chemical structure), whereas PCL is hydrophobic, being a polyester.

The small aggregates of complexes on the surface of the wall along the fibers are also well-evidenced by the rugosity of the wall, highlighted through height variations along three lines parallel to the axis fibers for each sample (see Figure 11).

Analyzing the height profile along the nanofiber surface in PCL sample (see Figure 11) it is evident how the height profile is smooth, with *R_a_* and *R_q_* values quite similar, confirming a very small roughness and the presence of very low peaks and valleys on the surface. In Figure 11, 1%AuM1 and 3%AuM1 height profiles become knurled, due to the presence of the complex aggregates, which causes an increase in the *R_a_* and *R_q_*. However, by the analysis of the local depth of the roughness, regardless of the concentration of the complex, the aggregates were found to have approximately the same size (30–70 nm).

#### 3.2.2. Structural Characterization of the Membranes

**Figure 12:**
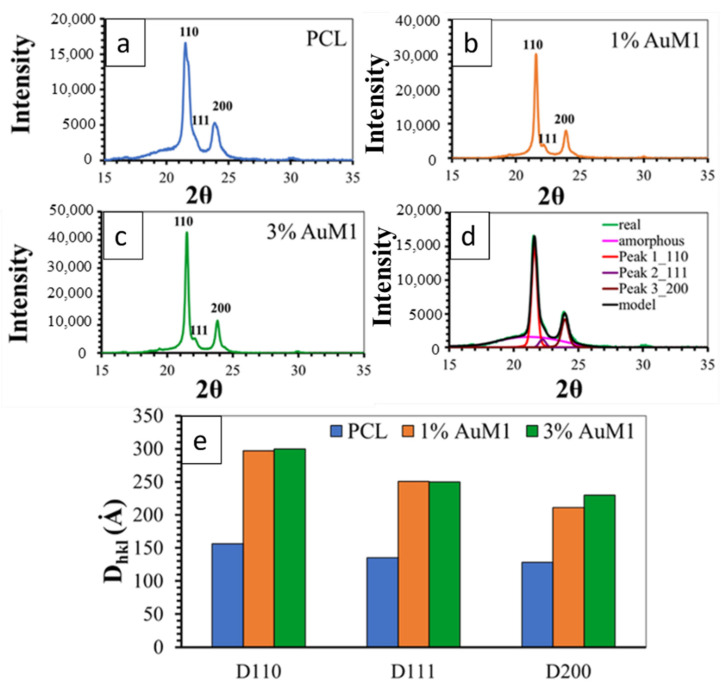
XRD spectra of (**a**) PCL; (**b**) 1%AuM1; (**c**) 3%AuM1; (**d**) Deconvolution Procedure and (**e**) crystallite size.

Figure 12 shows the XRD spectra of the unfunctionalized PCL membrane (Figure 12a) and 1%AuM1 (Figure 12b) and 3%AuM1 (Figure 12c) functionalized membranes. All analyzed membranes exhibit 2 intense peaks at 21.5° and 23.8° of 2θ corresponding to the (110) and (200) reflections, respectively, and the weaker peak at 22.1° of 2θ corresponding to the (111) reflection [34,44,53].

For the deconvolution of RX spectra, reported in Figure 12d, the maximum in the pattern of the amorphous halo was considered at a value of 2θ corresponding to 21°, as suggested in a previous paper [54]. The results of the deconvolution procedure are illustrated in Figure 12d.

Figure 12e shows the crystallite coherence lengths perpendicular to reflection planes 110 (D110), 200 (D200), and 111 (D111) for the PCL, 1%AuM1 and 3%AuM1 electrospun membranes. For the calculation of 110 (D110), 200 (D200) and 111 (D111), the half-height amplitude of the reflections was evaluated after the deconvolution of the RX spectra. The fraction of the crystalline phase (Xc) was calculated, after the deconvolution of the RX spectrum, as the ratio between the area under the crystalline peaks (excluding the area under the amorphous halo) and the area under the entire RX spectrum of the sample. The Xc of the analyzed membranes is shown in Table 4.

The diffractometric data highlight that the presence of the complex AuM1 affects crystallinity degree and crystal dimensions. The crystallinity increases with increasing the complex percentage (see Table 4). Concerning the size of the crystals, the fibers with the complex manifest a greater size of the crystals, while the percentage of the complex seems to have an almost negligible effect on this parameter (at least in the range of tested AuM1percentage). This result seems to confirm the morphological data discussed in the Section 3.2.1. Hence, the presence of the complex determines an increase both in the degree of crystallinity and size of the crystals. This difference in the crystallization behavior is most likely due to the kinetic of the solvent evaporation during the electrospinning process. Solvent evaporation of the PCL solution containing the complex is less rapid with respect to the solution without AuM1 complex; this creates greater mobility of the polymer segments before solidification of the membrane fibers on the collector-electrode. This confers the polymer chains during the crystallization phase, not only higher mobility but also a longer time to arrange themselves in the crystals and crystalline domains, causing crystals of higher size and higher crystallinity degree. This particular structural and morphological arrangement of the fibers/complex strongly affects the release profile of the complex from the membranes, as discussed in the next section.

#### 3.2.3. Encapsulation Efficiency and Drug Release

The analysis of the encapsulation efficiency was performed by dissolving the membranes in chloroform (CHCl_3_). In this way, by building a calibration line on the complex absorbance in chloroform, it is possible to evaluate the complex content after the complete dissolution of the membrane. Hence, knowing the complex content dissolved in the polymeric solution before the electrospinning process and evaluating the complex incorporated in the membrane, it is possible to evaluate the encapsulation efficiency of the process. It was also verified that the PCL absorbs at around 275 nm with a wide peak. For this reason, a PCL calibration line in chloroform was conducted, in order to subtract the PCL contribution to the peak of the gold complexes at 255 nm. This procedure has allowed obtaining the absorbance of the dissolved functionalized membranes. Then, the encapsulation efficiency was determined, as described in Equation (10).

(10) $$Encapsulation\;Efficiency\equiv \eta =\frac{m_{complex\;in\;the\;membrane}}{m_{theoretical\;complex}}\times 100$$

In Table 5, the encapsulation efficiencies of the functionalized membranes are shown.

These results highlight that the encapsulation efficiency is almost total (corresponding to the theoretical value) for the membranes at 1% of loading, whereas it ranges between 79% and 87% for the membranes loaded at 3%. The “theoretical value” is the total amount of complex solubilized in the solution before the electrospinning process. 

**Figure 13:**
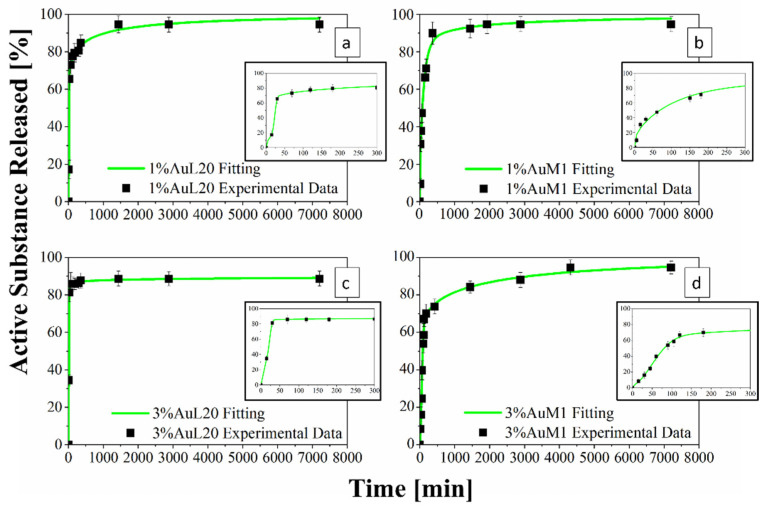
(**a**–**d**) Drug Release Curve and Modified Weibull Model for 1%AuL20 (**a**); 1%AuM1 (**b**); 3%AuL20 (**c**); and 3%AuM1 (**d**) functionalized membranes.

**Figure 14:**
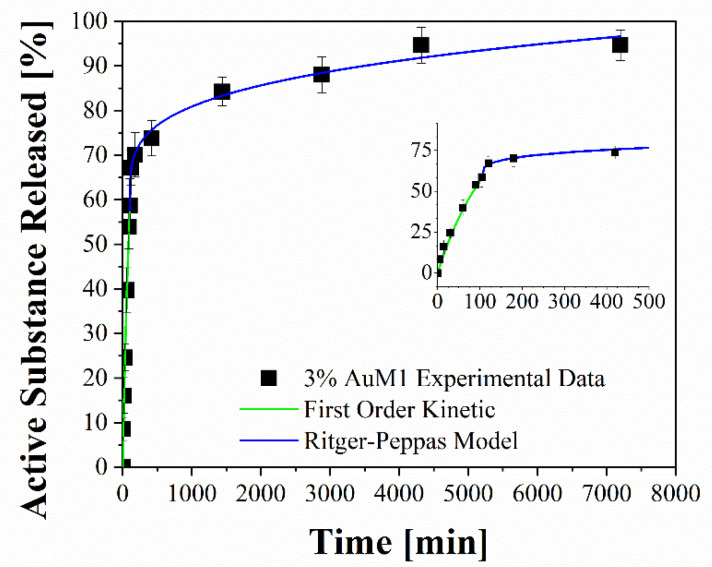
Fitting of experimental data through two-stage release model applied to 3%AuM1.

In light of these results, the authors have considered also taking into account the encapsulation efficiency in the drug release curves. For this reason, Figure 13 and Figure 14 were modified, defining the drug released as described in Equation (11):

(11) $$Active\;Substance\;Released\;\left[\%\right]=\frac{m\left(t\right)}{m_{theoretical\;complex}\times \frac{\eta }{100}}\times 100$$

where *m*(*t*) is the mass of the complex delivered in the medium at the time (*t*).

In this way, the drug release curves describe in an accurate way the profile of release referring to the real mass of complex encapsulated in the membrane.

#### 3.2.4. Drug Delivery Behavior and Experimental Data Modeling

Figure 13 shows the release profiles in the PBS medium, for the 1%AuL20 (a), 1%AuM1 (b), 3%AuL20 (c) and 3%AuM1 (d) functionalized membranes. It is well evidenced, from the profiles of all curves, that a large amount (~90% of the total released complex) of each active complex is released in the first 3 h. This result is fully in agreement with the peculiar morphological and structural organizations of the fibers constituting the membranes. In fact, since the active complex is consistently located on the nanofiber surface, as proven by AFM analysis, it is quickly released in the PBS medium in the first 3 h. The consistent burst is due to the dissolution of the external aggregates of active complexes that are directly in contact with the PBS solution. A very small percentage of complex is released in a longer time in an almost plateau region. This slower release is most likely due to the aggregate complexes entrapped among the crystals of the PCL fibers, which start to diffuse in the polymeric matrix toward the outer layer wall of the fibers. However, it is reasonable that in the very first steps, the second stage contribution is neglectable compared to the dissolution of drug present on the nanofiber surface. However, diffusive phenomena becomes considerable after the first hours of release. Coherently with XRD spectra, since the crystallinity and the crystalline size increase depending on the complex amount, a small decrease in the maximum amount release is recorded (from 98% to about 89% for AuL20, and from 95% to about 94% for AuM1) because the matrix ordered domains hinder the diffusion of the molecules through the nanofibers.

Experimental points in Figure 13 were fitted by using two different modeling approaches. The first is a modified Weibull model recently proposed in literature [31]. These types of models are generally applied to split the diffusion and the case II transport contribution [31,47]. However, this interpretation may not be appropriate in the presented systems, given the high burst expected because of the complex on the outer walls of the nanofibers. For this reason, in this case, the first part of the model describes the burst effect (*A*_1_, *b*_1_), whereas the second part (*A*_2_, *b*_2_) describes the diffusive effect. The fitting of the experimental data with the theoretical curves obtained from the modified Weibull model is shown in Figure 13 in a green color. In Table 6, the parameter values of the release models are reported.

To better analyze the contribution of the two release mechanisms, an approach similar to that proposed by Wu et al. [48] can be applied, which can be advantageously modified for interpreting the phenomena that occurs in the release of the complexes in our case. In particular, Wu et al. have modeled the drug release of their delivery system, splitting it into three zones and modeling each one appropriately depending on the release mechanism that occurs in that region.

Following this interesting approach, the two different behaviors in the release of the complex were considered in the present study. In particular, two regions are considered: the initial linear region corresponding to a very fast kinetic, and the subsequent region corresponding to the slowest kinetics. These two regions are fitted with two different models, since the drug release occurs because of different mechanisms. In particular, the first region, in which there is the dissolution of the external complex aggregates, was fitted by using a first-order model, since diffusive models are not suitable for describing the first part of a complex dissolution. The second zone of the curves was described by using a Ritger–Peppas model, which has confirmed for all membranes a diffusive mechanism (*n* < 0.45). The results are reported in Figure 14 and Table 7.

## 4. Conclusions

In this work, two synthesized complexes were proposed for potential application for contrasting malignant skin melanomas (MeWo cells). The Au-based complexes were incorporated in fibers of Polycaprolactone (PCL) membranes produced by the electrospinning technique. The complexes were also tested, for comparison, in the form of free complexes, directly in contact with the MeWo cell line culture. The tests performed on the functionalized membranes highlighted a very effective antitumoral activity in the first 24–28 h, which was very similar to that found for free complexes.

The peculiar chemical structure of the complexes, combined with the singular structural and morphological organization of the fibers constituting the functionalized electrospun membrane, determines a particularly favorable condition for having a very effective antitumor action in the first few hours of treatment. This favorable condition is due to the almost complete segregation of nanometric complex aggregates on the wall of the electrospun fibers and hence are promptly available for contact with the malignant cells. This occurrence clearly emerges from the Drug Release Curves, very fast in the initial stage, and the AFM analysis by which the evaluation of *R_a_* and *R_q_* parameters allowed for evaluating the presence of aggregates, of a few tens of nanometers, partially protruding from the walls.

Experimental points of the release profiles were perfectly fitted with theoretical curves, which easily allow for interpreting the kinetic phenomena occurring in the release of the complexes in the medium. From the theoretical curves emerge two different behaviors in the release of the complex. In particular, two regions were identified: the initial linear region corresponding to a very fast kinetic, and the subsequent region corresponding to a slowest kinetic. These two regions were fitted with two different models, as the drug release occurs because of different mechanisms. In particular, the first region, the dissolution of the complex aggregates, segregated on the fiber walls, was fitted by using a first-order model, since diffusive models are not suitable for describing the first part of the dissolution of the complex. In the second region, the behavior of the drug release was interpreted by using a Ritger–Peppas model, which confirms a diffusive mechanism for all the analyzed membranes. The overall results of the performed experimentation allow for foreseeing a relevant applicative potentiality. The functional membranes developed could be directly applied in the form of patches on the regions of the skin that needed anticancer treatment. Future research activities will be oriented toward this direction.

## Figures and Tables

**Table 1:** Process parameters.

Sample (Acronym)	Flow Rate [mL/h]	Distance Injector-Collector [cm]	Electric Potential Difference [kV]	Active Complex
PCL	2.0	28.5	21.0	0%
1%AuL20	2.0	22.5	25.0	1% of AuL20
3%AuL20	0.8	22.5	22.6	3% of AuL20
1%AuM1	1.0	25.0	24.0	1% of AuM1
3%AuM1	1.0	25.0	25.0	3% of AuM1

**Table 2:** Molar absorptivity coefficient of complexes.

#	*ε* [mL × mol^−1^ × cm^−1^]
**AuL20**	8.91
**AuM1**	4.98

**Table 3:** Pore size distribution parameters.

		PCL	1%AuM1	1%AuL20	3%AuM1	3%AuL20
**Pore Diameter**	**Mean [µm]**	1.16	0.99	2.59	1.22	2.17
	**Standard Deviation [µm]**	0.71	0.41	1.30	0.60	1.34

Coherently with literature, higher fiber dimensions induce higher pore sizes [50,51].

**Table 4:** Degree of crystallinity.

Crystalline Fraction (Xc)	
PCL	56.2%
1%AuM1	71.4%
3%AuM1	74.0%

**Table 5:** Encapsulation efficiency.

Membrane	η
1%AuL20	95.5%
1%AuM1	98.6%
3%AuL20	86.8%
3%AuM1	79.1%

**Table 6:** Modified Weibull Model parameters.

	Modified Weibull Parameters			
	1%AuM1	1%AuL20	3%AuL20	3%AuM1
**θ [-]**	0.533	0.522	0.562	0.544
$A_{1}\;[h^{b_{1}}]$	1.901	0.01030	0.01568	1.429
***b*_1_ [-]**	1.013	5.186	4.612	2.027
$A_{2}\;[h^{b_{2}}]$	1.276	1.793	0.8803	4.058
***b*_2_ [-]**	0.284	0.361	0.0429	0.461
***R* ^2^**	0.985	0.995	0.999	0.997

**Table 7:** Fitting parameters of drug release.

Sample	*R* ^2^	First Order Model	Ritger–Peppas Model	
		*a* [min^−1^]	*k* [min^−*n*^]	*n* [-]
1%AuM1	0.975	1.84 × 10^−2^	6.98 × 10^−2^	0.177
3%AuM1	0.990	8.18 × 10^−3^	3.83 × 10^−2^	0.259
1%AuL20	0.971	2.32 × 10^−2^	5.45 × 10^−2^	0.202
3%AuL20	0.988	3.90 × 10^−2^	4.84 × 10^−2^	0.101

## Data Availability

Not applicable.

## Supplementary Materials

The following supporting information can be downloaded at: www.mdpi.com/article/10.3390/ijms23137147/s1.

## References

[1] Kaidar-Person O., Bar-Sela G., Person B. (2011). The two major epidemics of the twenty-first century: Obesity and cancer. Obes. Surg..

[2] Epstein J.B., Thariat J., Bensadoun R.-J., Barasch A., Murphy B.A., Kolnick L., Popplewell L., Maghami E. (2012). Oral complications of cancer and cancer therapy. CA Cancer J. Clin..

[3] De Luna-Bertos E., Ramos-Torrecillas J., Garcia-Martinez O., Guildford A., Santin M., Ruiz C. (2013). Therapeutic doses of nonsteroidal anti-inflammatory drugs inhibit osteosarcoma MG-63 osteoblast-like cells maturation, viability, and biomineralization potential. Sci. World J..

[4] Cricchio V., Best M., Reverchon E., Maffulli N., Phillips G., Santin M., Della Porta G. (2017). Novel Superparamagnetic Microdevices Based on Magnetized PLGA/PLA Microparticles Obtained by Supercritical Fluid Emulsion and Coating by Carboxybetaine-Functionalized Chitosan Allowing the Tuneable Release of Therapeutics. J. Pharm. Sci..

[5] Moses M.A., Brem H., Langer R. (2003). Advancing the field of drug delivery: Taking aim at cancer. Cancer Cell.

[6] Zhong L., Li Y., Xiong L., Wang W., Wu M., Yuan T., Yang W., Tian C., Miao Z., Wang T. (2021). Small molecules in targeted cancer therapy: Advances, challenges, and future perspectives. Signal Transduct. Target. Ther..

[7] Feng X., Li J., Zhang X., Liu T., Ding J., Chen X. (2019). Electrospun polymer micro/nanofibers as pharmaceutical repositories for healthcare. J. Control. Release.

[8] Faramarzi N., Yazdi I.K., Nabavinia M., Gemma A., Fanelli A., Caizzone A., Ptaszek L.M., Sinha I., Khademhosseini A., Ruskin J.N. (2018). Patient-Specific Bioinks for 3D Bioprinting of Tissue Engineering Scaffolds. Adv. Healthc. Mater..

[9] Elkhoury K., Russell C.S., Sanchez-Gonzalez L., Mostafavi A., Williams T.J., Kahn C., Peppas N.A., Arab-Tehrany E., Tamayol A. (2019). Soft-Nanoparticle Functionalized Natural Hydrogels for Tissue Engineering Applications. Adv. Healthc. Mater..

[10] Paunescu V., Bojin F.M., Gavriliuc O.I., Taculescu E.A., Ianos R., Ordodi V.L., Iman V.F., Tatu C.A. (2014). Enucleation: A possible mechanism of cancer cell death. J. Cell. Mol. Med..

[11] Naves L.B., Dhand C., Venugopal J.R., Rajamani L., Ramakrishna S., Almeida L. (2017). Nanotechnology for the treatment of melanoma skin cancer. Prog. Biomater..

[12] Desoize B. (2004). Metals and metal compounds in cancer treatment—PubMed. Anticancer. Res..

[13] Orvig C., Abrams M.J. (1999). Medicinal Inorganic Chemistry: Introduction. Chem. Rev..

[14] Mora M., Gimeno M.C., Visbal R. (2019). Recent advances in gold–NHC complexes with biological properties. Chem. Soc. Rev..

[15] Gottlieb N.L. (1982). Comparative pharmacokinetics of parenteral and oral gold compounds. J. Rheumatol..

[16] Liu W., Gust R. (2012). Metal *N*-heterocyclic carbene complexes as potential antitumor metallodrugs. Chem. Soc. Rev..

[17] Hindi K.M., Panzner M.J., Tessier C.A., Cannon C.L., Youngs W.J. (2009). The Medicinal Applications of Imidazolium Carbene–Metal Complexes. Chem. Rev..

[18] Fung S.K., Zou T., Cao B., Lee P.Y., Fung Y.M.E., Hu D., Lok C.N., Che C.M. (2017). Cyclometalated Gold(III) Complexes Containing N-Heterocyclic Carbene Ligands Engage Multiple Anti-Cancer Molecular Targets. Angew. Chem. Int. Ed. Engl..

[19] Iacopetta D., Rosano C., Sirignano M., Mariconda A., Ceramella J., Ponassi M., Saturnino C., Sinicropi M.S., Longo P. (2020). Is the way to fight cancer paved with gold? Metal-based carbene complexes with multiple and fascinating biological features. Pharmaceuticals.

[20] Magherini F., Fiaschi T., Valocchia E., Becatti M., Pratesi A., Marzo T., Massai L., Gabbiani C., Landini I., Nobili S. (2018). Antiproliferative effects of two gold(I)-N-heterocyclic carbene complexes in A2780 human ovarian cancer cells: A comparative proteomic study. Oncotarget.

[21] Muenzner J.K., Biersack B., Albrecht A., Rehm T., Lacher U., Milius W., Casini A., Zhang J.J., Ott I., Brabec V. (2016). Ferrocenyl-Coupled N-Heterocyclic Carbene Complexes of Gold(I): A Successful Approach to Multinuclear Anticancer Drugs. Chemistry.

[22] Iacopetta D., Mariconda A., Saturnino C., Caruso A., Palma G., Ceramella J., Muià N., Perri M., Sinicropi M.S., Caroleo M.C. (2017). Novel Gold and Silver Carbene Complexes Exert Antitumor Effects Triggering the Reactive Oxygen Species Dependent Intrinsic Apoptotic Pathway. ChemMedChem.

[23] Welch D.R., Bisi J.E., Miller B.E., Conaway D., Seftor E.A., Yohem K.H., Gilmore L.B., Seftor R.E.B., Nakajima M., Hendrix M.J.C. (1991). Characterization of a highly invasive and spontaneously metastatic human malignant melanoma cell line. Int. J. Cancer.

[24] Liu X., Xu H., Zhang M., Yu D.G. (2021). Electrospun Medicated Nanofibers for Wound Healing: Review. Membranes.

[25] Longo R., Catauro M., Sorrentino A., Guadagno L. (2022). Thermal and mechanical characterization of complex electrospun systems based on polycaprolactone and gelatin. J. Therm. Anal. Calorim..

[26] Ashammakhi N., Ndreu A., Piras A.M., Nikkola L., Sindelar T., Ylikauppila H., Harlin A., Gomes M.E., Neves N.M., Chiellini E. (2006). Biodegradable Nanomats Produced by Electrospinning: Expanding Multifunctionality and Potential for Tissue Engineering. J. Nanosci. Nanotechnol..

[27] Mochane M.J., Motsoeneng T.S., Sadiku E.R., Mokhena T.C., Sefadi J.S. (2019). Morphology and Properties of Electrospun PCL and Its Composites for Medical Applications: A Mini Review. Appl. Sci..

[28] Alves Da Silva M.L., Martins A., Costa-Pinto A.R., Costa P., Faria S., Gomes M., Reis R.L., Neves N.M. (2010). Cartilage tissue engineering using electrospun PCL nanofiber meshes and MSCs. Biomacromolecules.

[29] Longo R., Gorrasi G., Guadagno L. (2021). Electromagnetically stimuli-responsive nanoparticles-based systems for biomedical applications: Recent advances and future perspectives. Nanomaterials.

[30] Longo R., Guadagno L., Lamberti P. Electromagnetic Characterization of Polycaprolactone electrospun nanofibers filled with Fe_3_O_4_ Nanoparticles. Proceedings of the 2020 4th International Symposium on Multidisciplinary Studies and Innovative Technologies (ISMSIT).

[31] Gorrasi G., Longo R., Viscusi G. (2020). Fabrication and Characterization of Electrospun Membranes Based on “Poly(ε-caprolactone)”, “Poly(3-hydroxybutyrate)” and Their Blend for Tunable Drug Delivery of Curcumin. Polymers.

[32] Zeng J., Yang L., Liang Q., Zhang X., Guan H., Xu X., Chen X., Jing X. (2005). Influence of the drug compatibility with polymer solution on the release kinetics of electrospun fiber formulation. J. Control. Release.

[33] Luraghi A., Peri F., Moroni L. (2021). Electrospinning for drug delivery applications: A review. J. Control. Release.

[34] Guadagno L., Raimondo M., Longo R., Sarno M., Iuliano M., Mariconda A., Saturnino C., Ceramella J., Iacopetta D., Sinicropi M.S. (2020). Development and characterization of antitumoral electrospun polycaprolactone/functionalized Fe_3_O_4_ hybrid membranes. Mater. Today Chem..

[35] Balashanmugam P., Sucharithra G., Agnes Mary S., Tamil Selvi A. (2020). Efficacy of biopolymeric PVA-AuNPs and PCL-Curcumin loaded electrospun nanofibers and their anticancer activity against A431 skin cancer cell line. Mater. Today Commun..

[36] Mariconda A., Sirignano M., Costabile C., Longo P. (2020). New NHC- silver and gold complexes active in A3-coupling (aldehyde-alkyne-amine) reaction. Mol. Catal..

[37] Palazzo I., Lamparelli E.P., Ciardulli M.C., Scala P., Reverchon E., Forsyth N., Maffulli N., Santoro A., Della Porta G. (2021). Supercritical emulsion extraction fabricated PLA/PLGA micro/nano carriers for growth factor delivery: Release profiles and cytotoxicity. Int. J. Pharm..

[38] Lamparelli E.P., Lovecchio J., Ciardulli M.C., Giudice V., Dale T.P., Selleri C., Forsyth N., Giordano E., Maffulli N., Della Porta G. (2021). Chondrogenic Commitment of Human Bone Marrow Mesenchymal Stem Cells in a Perfused Collagen Hydrogel Functionalized with hTGF-β1-Releasing PLGA Microcarrier. Pharmaceutics.

[39] Ciardulli M.C., Lovecchio J., Scala P., Lamparelli E.P., Dale T.P., Giudice V., Giordano E., Selleri C., Forsyth N.R., Maffulli N. (2021). 3d biomimetic scaffold for growth factor controlled delivery: An in-vitro study of tenogenic events on wharton’s jelly mesenchymal stem cells. Pharmaceutics.

[40] Scala P., Lovecchio J., Lamparelli E., Vitolo R., Giudice V., Giordano E., Selleri C., Rehak L., Maffulli N., Della Porta G. (2022). Myogenic commitment of human stem cells by myoblasts Co-culture: A static vs. a dynamic approach. Artif. Cells Nanomed. Biotechnol..

[41] De Winter J.C.F. (2019). Using the Student’s t-test with extremely small sample sizes. Pract. Assess. Res. Eval..

[42] Havlíček K., Svobodová L., Bakalova T., Lederer T. (2020). Influence of electrospinning methods on characteristics of polyvinyl butyral and polyurethane nanofibres essential for biological applications. Mater. Des..

[43] Naddeo C., Vertuccio L., Barra G., Guadagno L. (2017). Nano-Charged Polypropylene Application: Realistic Perspectives for Enhancing Durability. Materials.

[44] Sownthari K., Suthanthiraraj S.A. (2013). Synthesis and characterization of an electrolyte system based on a biodegradable polymer. Express Polym. Lett..

[45] Pavia D.L., Lampan G.M., Kriz G.S., Vyvyan J.R. (2015). Introduction to Spectroscopy.

[46] Romand J., Vodar B. (1951). Spectres d’absorption du benzene a l’etat vapeur et a l’etat condense dans l’ultraviolet lointain. Compt. Rend..

[47] Papadopoulou V., Kosmidis K., Vlachou M., Macheras P. (2006). On the use of the Weibull function for the discernment of drug release mechanisms. Int. J. Pharm..

[48] Wu J., Zhang Z., Gu J., Zhou W., Liang X., Zhou G., Han C.C., Xu S., Liu Y. (2020). Mechanism of a long-term controlled drug release system based on simple blended electrospun fibers. J. Control. Release.

[49] Zhan S., Wang J., Wang W., Cui L., Zhao Q. (2019). Preparation and in vitro release kinetics of nitrendipine-loaded PLLA–PEG–PLLA microparticles by supercritical solution impregnation process. RSC Adv..

[50] Ameer J.M., Anil Kumar P.R., Kasoju N. (2019). Strategies to tune electrospun scaffold porosity for effective cell response in tissue engineering. J. Funct. Biomater..

[51] Eichhorn S.J., Sampson W.W. (2005). Statistical geometry of pores and statistics of porous nanofibrous assemblies. J. R. Soc. Interface.

[52] Natu M.V., de Sousa H.C., Gil M.H. (2010). Effects of drug solubility, state and loading on controlled release in bicomponent electrospun fibers. Int. J. Pharm..

[53] Charitidis C.A., Dragatogiannis D.A., Milioni E., Kaliva M., Vamvakaki M., Chatzinikolaidou M. (2019). Synthesis, nanomechanical characterization and biocompatibility of a chitosan-graft-poly(ε-caprolactone) copolymer for soft tissue regeneration. Materials.

[54] Jeznach O., Kolbuk D., Sajkiewicz P. (2019). Aminolysis of Various Aliphatic Polyesters in a Form of Nanofibers and Films. Polymers.

