# Supplementary material for: Electrospun Membranes Designed for Burst Release of New Gold-Complexes Inducing Apoptosis of Melanoma Cells
Source: Int J Mol Sci. 2022 Jun 27;23(13):7147. doi: 10.3390/ijms23137147 (PMC9267035; doi:10.3390/ijms23137147)
Supplement: Supplementary file 1 [file ijms-23-07147-s001.zip › ijms-23-07147-s001/ijms-1742762-supplementary.pdf]

International Journal of *Molecular Sciences*

MDPI

Supplementary Materials

Electrospun Membranes designed for burst release of new Gold-Complexes inducing apoptosis of Melanoma cells

Liberata Guadagno[1,*], Marialuigia Raimondo[1], Luigi Vertuccio[2], Erwin Pavel Lamparelli[3], Maria Camilla Ciardulli[3], Pasquale Longo[4], Annaluisa Mariconda[5], Giovanna Della Porta[3,6] and Raffaele Longo[1,*]

[1] Department of Industrial Engineering, University of Salerno, Via Giovanni Paolo II 132, 84084 Fisciano (SA), Italy; rlongo@unisa.it (R. L.); mraimondo@unisa.it (M. R.); lguadagno@unisa.it; (L. G.)
[2] Department of Engineering, University of Campania "Luigi Vanvitelli", Via Roma 29, 813031 Aversa, Italy; luigi.vertuccio@unicampania.it (L. V.); michelina.catauro@unicampania.it (M. C.)
[3] Department of Medicine, Surgery and Dentistry, University of Salerno, Via S. Allende, 84081 Baronissi (SA), Italy; elamparelli@unisa.it (E. L.); mciardulli@unisa.it (M. C. C.); gdellaporta@unisa.it (G. D. P.)
[4] Department of Chemistry and Biology, University of Salerno, Via Giovanni Paolo II, 132, 84084 Fisciano, Italy; plongo@unisa.it (P. L.)
[5] Department of Science, University of Basilicata, Viale dell'Ateneo Lucano 10, 85100 Potenza, Italy; annaluisa.mariconda@unibas.it (A. M.)
[6] Interdepartment Centre BIONAM, Università di Salerno, Via Giovanni Paolo I, 84084 Fisciano (SA), Italy; gdellaporta@unisa.it
[*] Correspondence: rlongo@unisa.it (R. L.); lguadagno@unisa.it (L. G.)

1. Analysis of the synthesized complexes

1.1. Methods

NMR spectra were recorded on a Bruker AM 300 spectrometer (300 MHz for ^{1}H; 75 MHz for ^{13}C), a Bruker AVANCE 400 spectrometer (400 MHz for ^{1}H; 100 MHz for ^{13}C). NMR samples were prepared by dissolving about 15 mg of compounds in 0.5 ml of deuterated solvent. The ^{1}H NMR and ^{13}C NMR chemical shifts are given in ppm relative to $SiMe_4$ (δ=0 ppm) and they referenced to the residual impurities of the deuterated solvent signals (CD_2Cl_2: δ_H = 5.32 ppm, ^{13}C δ_c = 53.84 ppm – DMSO-d_6 δ_H = 2.50 ppm, ^{13}C δ_c = 39.52 ppm). Multiplicities are abbreviated as follows: singlet (s), doublet (d), triplet (t), multiplet (m).

Elemental analyses for C, H, and N were recorded with a Thermo-Finnigan Flash EA 1112 and were performed according to standard microanalytical procedures. Chloride was determined indirectly by reaction of $AgNO_3$ with halogen, precipitation of AgCl, which was dissolved in $Na_2S_2O_3$. The silver content in the solution was determined by flame atomic absorption spectroscopy (FAAS), and the halogen content was calculated by using the content of silver. Gold was determined with a burner (FIAS-100) air-acetylene flame. Solution of Au at a known concentration prepared from a stock solution of 1 g/l (Carlo Erba, Milan, Italy) was used as standards. The instrument was set at zero using a 1% solution of HNO_3. Sample scripts were analyzed along with their white.

Mass spectrum of AuL20 was acquired with a Bruker solariX Fourier transform mass spectrometer (Bruker Daltonik GmbH, Bremen, Germany) equipped with a 7 T refrigerated actively shielded superconducting magnet (Bruker Biospin, Wissembourg, France). The sample was ionized in negative ion mode using the ESI ion source (Bruker Daltonik GmbH, Bremen, Germany). Sample solution was continuously infused using a syringe at a flow rate of 120 μL/h. The detection mass range was set to m/z 150 – 3000. The mass spectrum was calibrated externally with NaTFA clusters in negative ion mode using a linear calibration. The source voltage was set to -3.9 kV, the dry gas (nitrogen) flow rate to 4L/min at 200 °C.

MALDI-MS: mass spectrum was acquired using a Bruker SolariX XR Fourier transform ion cyclotron resonance mass spectrometer (Bruker Daltonik GmbH, Bremen,

Germany) equipped with a 7 T refrigerated actively-shielded superconducting magnet (Bruker Biospin, Wissembourg, France). The sample was ionized in positive ion mode using the MALDI ion source (Bruker Daltonik GmbH, Bremen, Germany). The mass range was set to *m*/*z* 200 – 3000. The laser power was 28% and 22 laser shots were used for each scan. The mass spectra were calibrated externally using a mix of peptide clusters in MALDI ionization positive ion mode. A linear calibration was applied. To improve the mass accuracy, the sample spectra were recalibrated internally by matrix ionization (2,5-dihydroxybenzoic acid).

1.2. Results

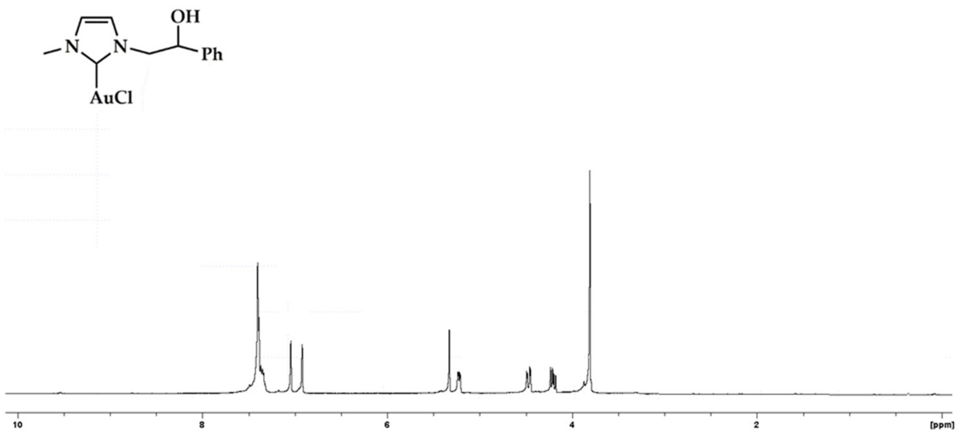

Figure S1. ^{1}H NMR (300 MHz, CD_2Cl_2, δ ppm): 7.41-7.35 (m, 5H, *Ph ring*); 7.05 (d, 1H, NC*H*CHN); 6.92 (d, 1H, CHC*H*N); 5.22 (t, 1H, C*H*OH); 4.47-4.21 (dd, 2H, NC*H*$_2$); 3.80 (s, 3H, NC*H*$_3$).

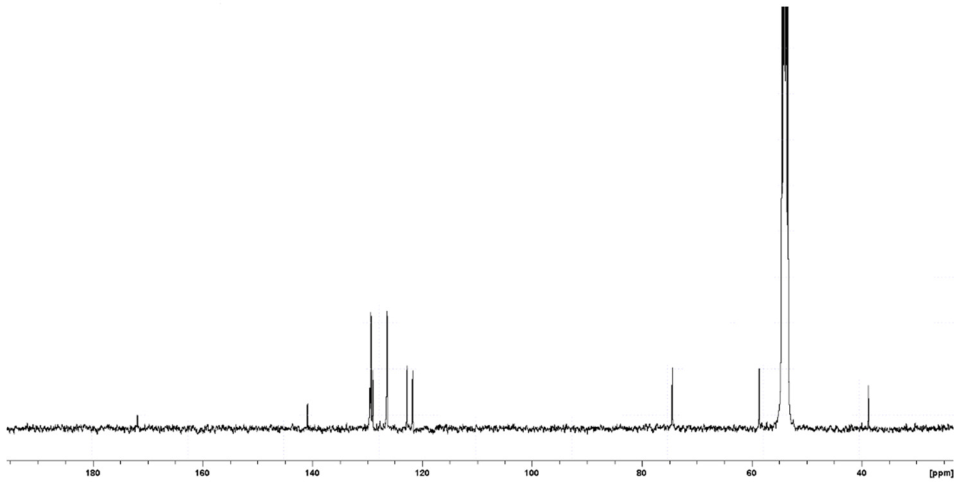

Figure S2. ^{13}C NMR (100 MHz, CD_2Cl_2, δ ppm): 171.8 (NCN); 140.9, 129.3, 129.0, 126.5, (*Ph ring*); 122.7 (NCHCHN), 121.7 (NCHCHN), 74.5 (CHOH), 58.5 (NCH_2), 38.8 (NCH_3).

Elemental analysis: found (%): C 33.25; H 3.24; Au 45.40; Cl 8.01; N 6.25; O 3.53. Calc. For $C_{12}H_{14}AuClN_2O$ (%): C 33.16; H 3.25; Au 45.31; Cl 8.16; N 6.44; O 3.68.

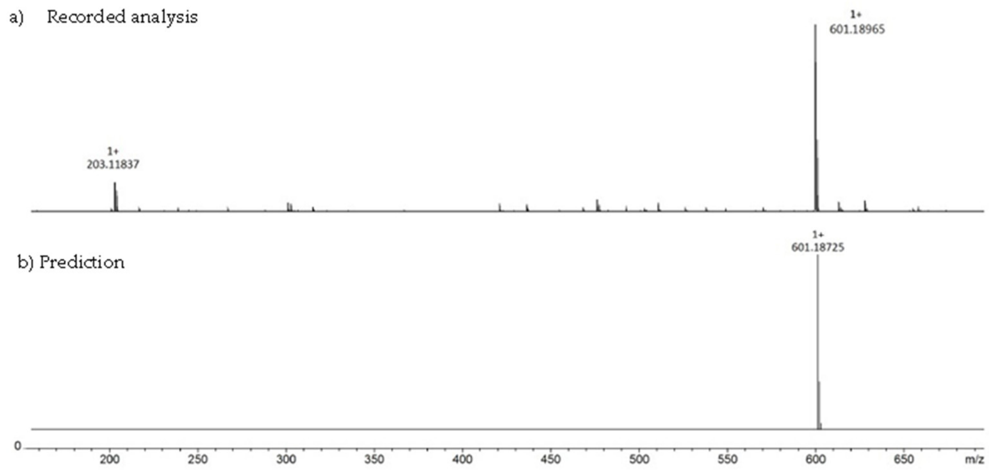

Figure S3. ESI-MS (CH_2Cl_2, *m/z*): 601.18965 Dalton $[Au(NHC)_2]^+$ and 203.11837 Da attributable to $[C_{12}H_{15}N_2O]^+$.

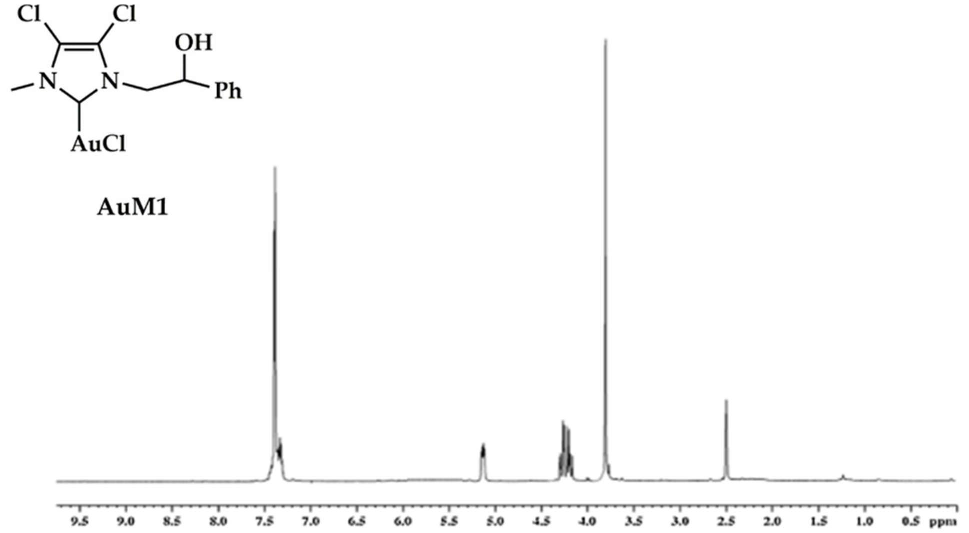

Figure S4. ^{1}H NMR (400 MHz, DMSO-d_6, δ ppm): 7.33 (m, 5H, *Ph ring*), 5.13 (m, 1H, C*H*OH), 4.23 (dd, 2H, NC*H*$_2$CHOH), 3.82 (s, 3H, C*H*$_3$).

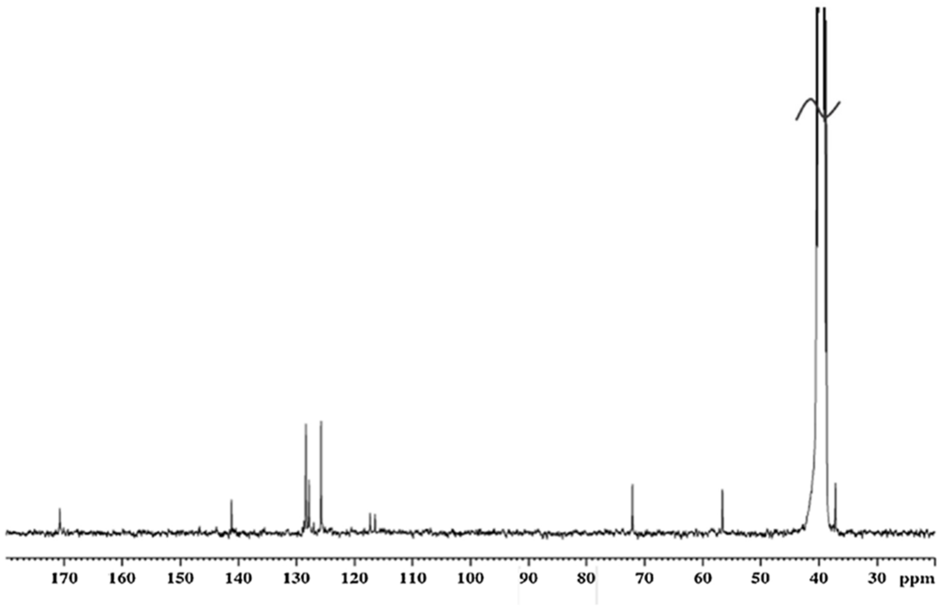

Figure S5. ^{13}C NMR (75 MHz, DMSO-d_6, δ ppm): 170.7 (NCN), 141.2, 128.4, 127.8, 127.0, 125.7 (aromatic carbons), 117.3 and 116.4 (NCClCClN), 72.0 (CH_2CHOH), 56.6 (NCH_2), 37.1 (NCH_3).

Elemental analysis: found (%): C 28.59; H 2.47; Au 39.21; Cl 21.24; N 5.45; O 3.27. Calc. For $C_{12}H_{12}AuCl_3N_2O$ (%): C 28.62; H 2.40; Au 39.12; Cl 21.12; N 5.56; O 3.18.

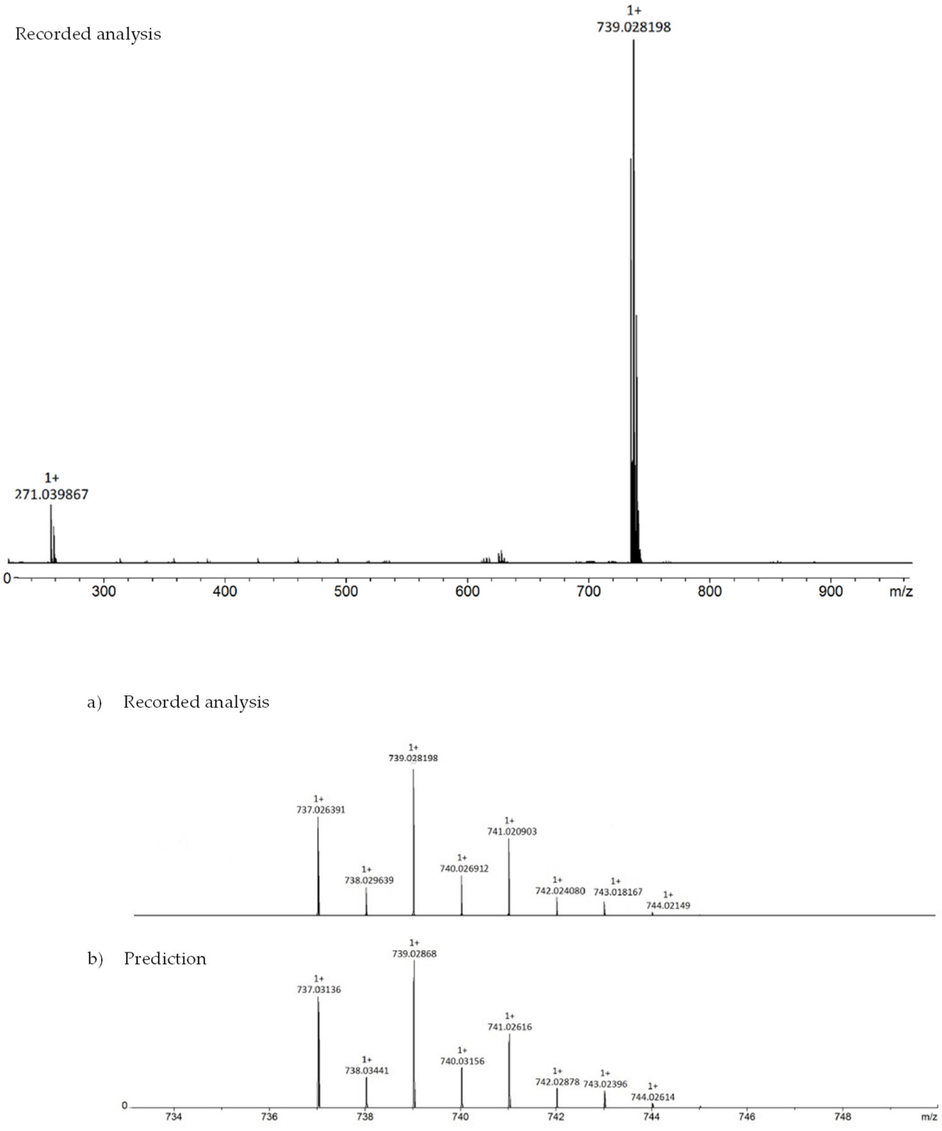

Figure S6. MALDI-MS (CH_2Cl_2, *m/z*): 739.028198 Da attributable to $[Au(NHC)_2]^+$ and 271.039867 Da attributable to $[C_{12}H_{13}Cl_2N_2O]^+$.

2. Results of the tests on cell viability performed with unloaded membranes

Results on tests performed with the unloaded membranes are shown in Figure S7.

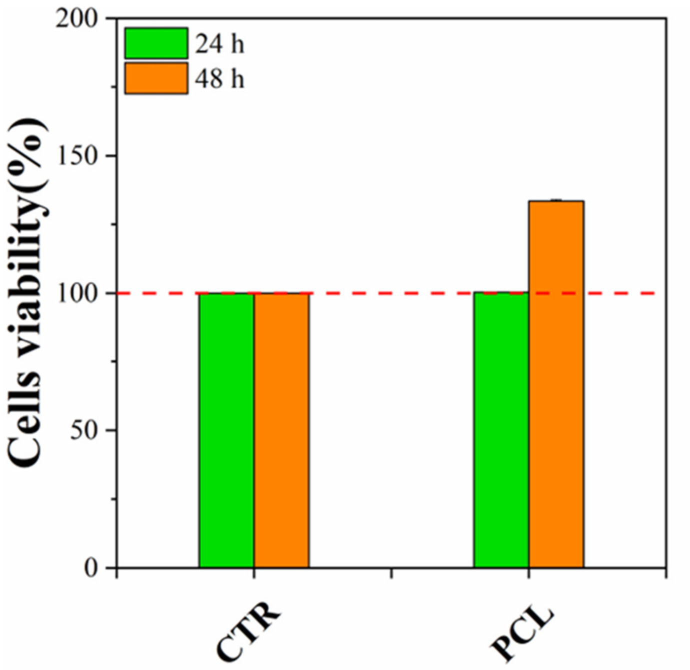

Figure S7. MTT assay on MeWo cells when cultured on unfunctionalized PCL membranes surface for 24h and 48h.
